# Fisheries genomics of snapper (*Chrysophrys auratus*) along the west Australian coast

**DOI:** 10.1111/eva.13439

**Published:** 2022-07-09

**Authors:** Andrea Bertram, David Fairclough, Jonathan Sandoval‐Castillo, Chris Brauer, Anthony Fowler, Maren Wellenreuther, Luciano B. Beheregaray

**Affiliations:** ^1^ Molecular Ecology Laboratory, College of Science and Engineering Flinders University Adelaide SA Australia; ^2^ Aquatic Sciences and Assessment Department of Primary Industries and Regional Development Perth WA Australia; ^3^ Aquatic Sciences South Australian Research and Development Institute Adelaide SA Australia; ^4^ The New Zealand Institute for Plant and Food Research Limited Nelson New Zealand; ^5^ The School of Biological Sciences University of Auckland Auckland New Zealand

**Keywords:** ddRADseq, fisheries management, marine connectivity, marine teleost, population genomics, stock structure

## Abstract

The efficacy of fisheries management strategies depends on stock assessment and management actions being carried out at appropriate spatial scales. This requires understanding of spatial and temporal population structure and connectivity, which is challenging in weakly structured and highly connected marine populations. We carried out a population genomics study of the heavily exploited snapper (*Chrysophrys auratus*) along ~2600 km of the Australian coastline, with a focus on Western Australia (WA). We used 10,903 filtered SNPs in 341 individuals from eight sampling locations to characterize population structure and connectivity in snapper across WA and to assess if current spatial scales of stock assessment and management agree with evidence from population genomics. Our dataset also enabled us to investigate temporal stability in population structure as well as connectivity between WA and its nearest, eastern jurisdictional neighbour. As expected for a species influenced by the extensive ocean boundary current in the region, low genetic differentiation and high connectivity were uncovered across WA. However, we did detect strong isolation by distance and genetic discontinuities in the mid‐west and south‐east. The discontinuities correlate with boundaries between biogeographic regions, influenced by on‐shelf oceanography, and the sites of important spawning aggregations. We also detected temporal instability in genetic structure at one of our sites, possibly due to interannual variability in recruitment in adjacent regions. Our results partly contrast with the current spatial management of snapper in WA, indicating the likely benefits of a review. This study supports the value of population genomic surveys in informing the management of weakly structured and wide‐ranging marine fishery resources.

## 1 INTRODUCTION

Marine ecosystems are one of the last on the planet in which wild populations are heavily exploited for human consumption. Not only do aquatic animals constitute ~17% of the world’s meat consumption, but their capture supports the livelihoods of an estimated 10–12% of the global population and many species hold cultural significance (FAO, 2020). Although more than half of all assessed fish stocks were likely depleted by the mid‐1970s, the proportions of depleted stocks recently dropped to below one quarter in regions practising intensive evidence‐based fisheries management (FAO, 2020; Hilborn et al., 2020). Indeed, the intensity of management efforts and knowledge of species biology strongly correlate with fisheries sustainability and therefore realized fisheries yield (Hilborn et al., 2020; Nilsson et al., 2019). Knowledge of biological stock structure (i.e. population structure), defined as the number and spatial extent of distinct populations within a species' range, as well as the levels of connectivity between such stocks, is essential for the performance of fishery assessments and sustainable management (Cadrin, 2020). This is because distinct stocks may display different dynamics (e.g. recruitment, growth rates), demographics (e.g. sex ratios, abundance) and genetics (e.g. diversity, environmental adaptations), causing unique responses to fishing and environmental pressures (Cadrin et al., 2020). The consequences of not incorporating accurate information on stock structure and connectivity in assessment and management have been widely documented (Fu & Fanning, 2004; Kell et al., 2009; Kerr et al., 2014; Smedbol & Stephenson, 2001; Sterner, 2007; Ying et al., 2011). For example, consideration of the complex stock structure of Bristol Bay sockeye salmon (*Oncorhynchus nekra*) has been important for maintaining fishery productivity, while depletions have occurred in other places where spatial structure has not been managed effectively (Hilborn et al., 2003; Schindler et al., 2010).

Key biological traits that shape population structure and connectivity in marine species include: habitat specialization, determined by breeding site preferences, dietary requirements and abiotic tolerance limits (Cowen, 2002; Cowen & Sponaugle, 2009); and dispersal potential (Bohonak, 1999), determined by reproductive strategy (e.g. broadcast spawning and brooding), the duration of any pelagic stages and the movement behaviour of both larval and postlarval stages. Due to the fluidity of the marine environment and processes like currents, waves and tides, connectivity and population homogeneity can occur over large spatial scales, particularly in species with long pelagic larval stages (PLS; Cowen & Sponaugle, 2009; Selkoe et al., 2008). For example, in the broadcast spawning and highly migratory yellowfin tuna (*Thunnus albacares*), population homogeneity likely occurs at spatial scales as extensive as entire ocean basins (Barth et al., 2017; Pecoraro et al., 2018). Nevertheless, marine environments are dynamic and heterogeneous and therefore contain elements that can disrupt connectivity and act as barriers to dispersal, including meanders and eddies, fronts, irregular coastline topology, habitat heterogeneity and countercurrents (Cowen, 2002; Cowen & Sponaugle, 2009). For example, Taillebois et al. (2017) detected significant population structuring in the broadcast spawning marine teleost, the black‐spotted croaker (*Protonibea diacanthus*), across topographically complex coastline in northern Australia at spatial scales of only 100 s of kilometres.

Methods for assessing stock structure and connectivity in exploted marine species include otolith microchemistry, isotope analysis, mark‐recapture and DNA analyses. Advances in DNA sequencing technologies have increased the value of DNA‐based methods in stock delineation studies, and it is now economical to generate large datasets of 1000 s of DNA markers (i.e. those used in genomic studies), vastly contrasting with previously utilized datasets of 10 s of markers (i.e. those used in microsatellite studies). Large datasets of DNA markers have greater resolving power to uncover the often subtle patterns of population structure and connectivity characteristic of marine species (Bernatchez et al., 2017; Grummer et al., 2019), occurring due to their high abundances and dispersal abilities.

**FIGURE 1:**
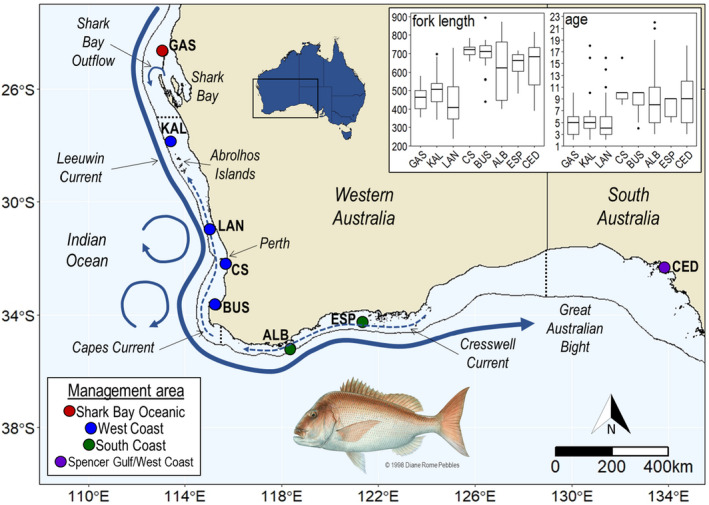
Map of the study region showing the eight sampling sites, including the comparative site in South Australia (SA), the three current management areas for snapper in Western Australia (WA): Shark Bay Oceanic (red), West Coast (blue) and South Coast (green), as well as the westernmost management area in SA: Spencer Gulf/West Coast (purple). Management area boundaries are indicated with dotted black lines. The inset plots summarize the lengths (in mm) and ages (in years) of sampled snapper (excluding the temporal samples from CS and ESP). Sampling sites in WA: GAS, Gascoyne; KAL, Kalbarri; LAN, Lancelin; CS, Cockburn Sound; BUS, Busselton; ALB, Albany; ESP, Esperance. Sampling site in SA: CED, Ceduna

Our focus here is on snapper (*Chrysophrys auratus*), a large, long‐lived demersal sparid distributed in subtropical and temperate coastal waters of Australia and New Zealand (Gomon et al., 2008). In Western Australia (WA), snapper supports highly valuable commercial and recreational fisheries from the Shark Bay region to the border of South Australia (SA; Figure 1). In 2017/18, snapper production by the WA commercial sector was valued at ~1.7 million AUD (Steven et al., 2021), while snapper constituted ~4% of catch by the WA recreational sector (Ryan et al., 2019), an industry that generates ~2.4 billion AUD per year (McLeod & Lindner, 2018). During 2017/2018, ~242 tonnes of snapper were landed in WA state waters across all fishing sectors (of which ~50% was commercial catch), with the largest catches landed off the west coast (Gaughan & Santoro, 2020; Ryan et al., 2019). Due to its fishery importance, its economic and social importance, and its inherent vulnerability to fishing as a result of its biology (e.g. long‐lived, aggregate spawner), snapper is used as an indicator species for the inshore suite of demersal scalefish resources across its WA range (Newman et al., 2018). In WA, snapper is assessed and managed as three separate stocks (not including the three stocks in the inner gulfs of Shark Bay not addressed in this study)—the Shark Bay Oceanic, West Coast and South Coast stocks (Figure 1). The Shark Bay Oceanic and West Coast stocks are currently considered depleted, with management in place for recovery, while the South Coast stock is considered sustainable (Fairclough et al., 2021; Newman et al., 2022). These stocks and their spatial boundaries are based on bioregions defined primarily from information on environmental characteristics rather than from knowledge of species‐specific population structure and stock connectivity (Newman et al., 2022).

The particular reproductive characteristics and movement behaviours of snapper as well as the dominant patterns of oceanographic circulation along the WA coast are likely important factors for shaping stock structure and connectivity in the species. The dispersal potential of snapper is expected to be high, particularly during the pelagic and subadult stages (Fairclough et al., 2013; Parsons et al., 2014). Snapper are multiple batch, broadcast spawners, with eggs hatching after 1–2 days and the pelagic larval stage lasting for ~17–30 days (Fowler & Jennings, 2003; Francis, 1994). When eggs and larvae occur in open waters, they are expected to be influenced by the Leeuwin Current (LC) and the seasonal, wind‐driven Capes and Cresswell Currents (see Figure 1, Akhir et al., 2020; Cresswell & Golding, 1980; Cresswell & Peterson, 1993; Pearce & Pattiaratchi, 1999). After settlement, juvenile snapper migrate from spawning areas to sheltered inshore habitats where they reside until ~2 years of age. During the subadult stage, snapper are known to migrate distances of more than 1000 km to the coastal regions where they become resident (Fowler et al., 2005; Hamer et al., 2011; Wakefield et al., 2011).

Other life‐history and oceanographic characteristics could act to limit connectivity and dispersal in snapper in WA, leading to population structure. Although snapper spawn along the entire WA coastline, a number of embayments where large spawning aggregations occur are particularly important (Nahas et al., 2003; Wakefield, 2010; Wakefield et al., 2015). These include coastal embayments of Shark Bay, Perth (Cockburn Sound, Warnbro Sound, Owen Anchorage) and Albany (King George Sound; Figure 1). These environments are largely protected from oceanographic processes in open shelf waters and also often feature local circulation patterns that act to retain eggs and larvae (Steedman & Craig, 1983; Wakefield et al., 2011). With respect to older life‐stages, although adult snapper are known to travel distances of hundreds of kilometres or more, most show more limited movements of spatial scales of <100 km (Crisafulli et al., 2019; Moran et al., 2003; Sumpton et al., 2003).

Current knowledge of population structure and connectivity in snapper in WA is based on tagging, mark‐recapture, otolith microchemistry and microsatellite analyses. Recent mark‐recapture work on the lower west coast of WA indicated that adult movement is largely limited to spatial scales of <20 km, although this estimate is probably influenced by spatial variability in fishing effort (Crisafulli et al., 2019). Microsatellite DNA analyses have suggested that snapper in WA are characterized by an isolation by distance pattern of population structure, rather than genetically distinct subpopulations (Gardner & Chaplin, 2011). Otolith microchemistry studies have reported different patterns of mixing during different life stages as well as a range of patterns of spatial differentiation using different chemical tags (Edmonds et al., 1999; Fairclough et al., 2013). Additionally, the latter study (i.e. Fairclough et al., 2013) demonstrated that adults in any one location are derived from multiple nearshore nursery environments. Therefore, a degree of uncertainty still remains around where appropriate stock boundaries should be drawn for management and assessment purposes and whether current boundaries are appropriate. In addition, declines in snapper catches and stock depletions in parts of WA point to the need for more information about population structure and connectivity across the state (Fowler et al., 2021).

In this study, we use genome‐wide polymorphism data and population genomic analyses to characterize population genetic structure and connectivity in snapper across its WA range. Our dataset also enables us to assess temporal stability in population structure as well as connectivity in snapper between WA and its nearest, eastern jurisdictional neighbour (SA). Given the species’ life‐history traits and the oceanographic setting of WA's coast, we predict population differentiation in snapper to be influenced by broad patterns of on‐shelf oceanographic circulation. Our secondary goal is to determine whether current spatial scales of assessment and management reflect the biological units (i.e. stocks) identified with genomics, as well as their spatial boundaries. Our third goal is to demonstrate to fisheries scientists and managers the value of genomic datasets in clarifying stock structure and connectivity in a species for which this was previously difficult (i.e. highly abundant and dispersive species using smaller genetic datasets).

## 2 METHODS AND MATERIALS

### 2.1 Sampling and associated biological information

Muscle or fin‐clip samples were obtained from 290 snapper from eight locations between the Gascoyne, on the central west coast of Western Australia (WA), and Ceduna, on the west coast of South Australia (SA; see Figure 1, Table 1 and Table S1). Tissue samples were obtained from fish landed by recreational or commercial fishermen or fisheries researchers (as part of fisheries independent research surveys) between 2018 and 2020 during routine sampling for stock assessment by the Department of Primary Industries and Regional Development (DPIRD) WA and the South Australia Research and Development Institute. The eight sampling locations cover the majority of fishing activity occurring in our focal jurisdiction WA and include both commercial hotspots (Gascoyne, Kalbarri and Lancelin) and recreational fishing hotspots near population centres (Cockburn Sound, Busselton and Albany; Newman et al., 2022). Our localities also cover the management areas used for snapper in WA outside of the inner gulfs of Shark Bay (not addressed here) – Shark Bay Oceanic, West Coast and South Coast (see Figure 1). Tissues were preserved in 100% ethanol and stored at −20°C until DNA extraction. Where possible, biological data including total and fork length, age, sex and reproductive stage were obtained for each sampled individual (see Table 1 and Table S1).

**TABLE 1:** Levels of genome‐wide variation for all snapper samples from the seven Western Australian locations (including the two temporal samples from Cockburn Sound and Esperance: CS14 and ESP10) and the comparative South Australian site (CED), based on 10,903 putatively neutral SNPs

Site	*N*	Avg. FL	Avg. age	*H*_O_	*H*_E_	%PL	*F*_IS_
Gascoyne (GAS)	40 (39)	457.4 (61.5)	5.1 (2.0)	0.181	0.185	92.5	0.029
Kalbarri (KAL)	40 (40)	497.3 (84.8)	5.7 (3.5)	0.182	0.186	92.8	0.025
Lancelin (LAN)	37 (37)	441.6 (130.8)	5.1 (3.3)	0.184	0.186	93.3	0.020
Cockburn Sound (CS)	40 (39)	713.5 (30.0)	9.7 (0.6)	0.186	0.187	94.1	0.012
Cockburn Sound 2014 (CS14)	30 (29)	794.9 (42.7)	N/A	0.180	0.184	90.3	0.016
Busselton (BUS)	40 (40)	702.8 (72.1)	9.0 (1.5)	0.180	0.185	94.5	0.030
Albany (ALB)	40 (39)	611.1 (163.0)	8.9 (5.5)	0.189	0.188	95.3	0.008
Esperance (ESP)	13 (13)	631.8 (92.9)	7.6 (1.9)	0.187	0.182	80.3	0.007
Esperance 2010 (ESP10)	28 (28)	N/A	N/A	0.184	0.186	91.7	0.015
Ceduna (CED)	37 (37)	634.2 (127.2)	8.7 (4.1)	0.188	0.187	95.0	0.005

*Note*: *N*, sample size before and after (in parentheses) removing individuals with >20% missing data; No *F*_IS_ value deviated significantly from expectation (α = 0.05). Mean fork length (FL) is in mm and age in years. Following each mean FL and age is the associated standard deviation in parentheses.

Abbreviations: %PL, per cent polymorphic loci; *F*_IS_, inbreeding coefficient; FL, fork length; *H*e, expected heterozygosity; *H*o, observed heterozygosity.

Additional tissue samples were obtained from snapper landed off Esperance (*n* = 28) and in Cockburn Sound (*n* = 30) in 2010 and 2014, respectively, to investigate the temporal stability of trends in population structure. Although the time periods covered preclude investigation across multiple generations, they are adequate for assessing interpopulation migration and population range shifts as a result of temporal variation in recruitment or population density (information important in fisheries management). The Cockburn Sound samples were collected by DPIRD WA staff as part of a fisheries independent survey, while the Esperance samples were collected by DPIRD WA staff from commercial catch for a former population genetics project (see Gardner et al., 2014, 2017; Gardner & Chaplin, 2011).

### 2.2 DNA extraction, genomic library preparation and sequencing

Genomic DNA was isolated from each sample using a modified salting‐out protocol (Sunnucks & Hales, 1996). Quality control of DNA extracts was carried out with NanoDrop and gel electrophoresis. Extracts were quantified with Qubit and diluted to ~10–15 ng/μl. Double‐digest restriction site‐associated DNA (ddRAD) libraries were constructed following a protocol modified from Peterson et al. (2012), as detailed in Brauer et al. (2016). For each sample, 200 ng of genomic DNA was digested using the restriction enzymes Sbfl‐HF and Msel (New England Biolabs). One of 96 unique 6‐bp barcodes was ligated to each sample before pooling libraries into groups of 12 samples. DNA fragments between 300 and 800 bp were selected from each pool using a Pippin Prep (Sage Science). Each pool was then amplified in three 25 μl reactions to reduce PCR artefact bias. Following PCR, the three reactions were combined, and the size distribution of the products examined using a 2100 Bioanalyser (Agilent Technologies) and quantified using Qubit. Aliquots of equal concentrations were then taken from each pool and combined to form one pool of 96 samples. Pools were sequenced on four lanes of an Illumina HiSeq 4000 (150 bp paired end) at Novogene (Hong Kong). Six replicates were included in each pool of 96 samples so that sequencing and genotyping errors could be quantified.

### 2.3 Bioinformatics

Raw sequence reads were processed to generate a high‐quality SNP dataset using similar bioinformatic procedures as detailed elsewhere (Sandoval‐Castillo et al., 2018), but with the assistance of a reference genome for snapper. Specifically, the quality of raw sequence data was checked using FastQC before being demultiplexed with the process_radtags module from STACKS 2.0 (Catchen et al., 2013). Barcodes, restriction sites and RAD tags were then trimmed from sequence reads using TRIMMOMATIC (Bolger et al., 2014). Trimmed sequence reads were then aligned to a high‐quality snapper reference genome (Catanach et al., 2019) using BOWTIE 2 (Langmead & Salzberg, 2012). The SNPs were subsequently called using BCFTOOLS (Narasimhan et al., 2016). The resulting dataset was initially filtered using VCFTOOLS (Danecek et al., 2011) to retain only bi‐allelic SNPs present in at least 80% of individuals in all populations with a minimum minor allele frequency of 0.03. Also using VCFTOOLS, further filtering was carried out to remove indels, individuals with more than 20% missing data, SNPs with low and extremely high coverage, SNPs with low mapping quality, SNPs not in Hardy–Weinberg Equilibrium (HWE) and physically linked SNPs.

### 2.4 Categorizing putatively neutral SNPs

The Bayesian method in BAYESCAN 3.0 (Foll & Gaggiotti, 2008) was used to identify candidate SNPs putatively under selection. The software was run with 20 pilot runs, each with 5000 iterations, followed by 100,000 iterations with a burn‐in length of 50,000 iterations. The outlier SNPs were identified using a 5% false discovery rate with a prior odd of 10 and were subsequently removed from the dataset to produce a putatively neutral one. The study of the role of natural selection on snapper populations using outlier SNPs and genomic regions associated with environmental variation is the topic of a separate and ongoing investigation (Brauer et al. unpublished).

### 2.5 Genetic diversity, population differentiation and clustering analyses

The genetic diversity statistics observed heterozygosity (*H*_O_), expected heterozygosity (*H*_E_) and per cent polymorphic loci (%PL), were calculated with the *populations* module of STACKS 2 (Rochette et al., 2019). Pairwise *F*_ST_ and population specific *F*_IS_ values were calculated in ARLEQUIN 3.5 (Excoffier & Lischer, 2010), with significance assessed with 1000 permutations. *F*_ST_ associated *p*‐values were subsequently corrected for multiple comparisons through the false discovery rate (FDR) method using the *p.adjust* function in the R package BASE 4.0.3 (Team, 2021). Global *F*_ST_ was determined using the R package HIERFSTAT 0.5–10 (Goudet et al., 2015). The model‐free approach, Principal Components Analysis (PCA), was then carried out using VEGAN 2.5‐6 (Oksanen et al., 2018) in R. Missing genotypes (~0.6% of data matrix) were assigned with the most common genotype at that locus. A discriminant analysis of principal components (DAPC) was also conducted using the R package ADEGENET 2.1.5 (Jombart, 2008; Jombart et al., 2010) to assess consistency with PCA groupings. DAPC is similar to PCA except that it aims to maximize between group variation while minimizing differentiation within groups. Population structure was further assessed with the maximum likelihood approach implemented in ADMIXTURE 1.3 (Alexander et al., 2009; Alexander & Lange, 2011). The cross‐validation procedure in ADMIXTURE was employed to determine the most likely *K* value. To do this, a 5‐fold cross‐validation was performed for *K* values 1–8. Graphical representation of population assignments was performed with GGPLOT2 3.3.3 (Wickham, 2016) in R. ADMIXTURE was run with and without the SA sample (CED) to assess its effect on analysis outcomes. To assess temporal stability in patterns of population structure, the ADMIXTURE analysis was also carried out including the two temporal samples and membership to any identified clusters was compared between sampling periods. Finally, we carried out an analysis of molecular variance (AMOVA) in ARLEQUIN 3.5 (Excoffier & Lischer, 2010) to assess partitioning of genetic variation at different heirachical levels, with groups assigned according to the results of PCA and ADMIXTURE and significance determined with 1000 permutations.

### 2.6 Isolation by coastal distance

We tested for a signal of isolation by distance (IBD) across the sampling range by assessing the relationship between coastal distance and linearized *F*_ST_ (*F*_ST_/[1−*F*_ST_]) with a Mantel test (9999 permutations) in GENALEX 6.5 (Peakall & Smouse, 2012). Distances between sampling locations were estimated as the shortest distance between sites following the coastline using the *viamaris* function in MELFUR 0.9 (https://github.com/pygmyperch/melfuR). Mean effective dispersal distance was then estimated from the slope of the IBD relationship using the theoretical model of Kinlan and Gaines (2003), an extension of Palumbi (2003): mean dispersal distance = 0.0016(IBD slope)^−1.0001^. The resulting estimate equates to the mean dispersal distance required to produce the observed IBD slope under model assumptions about parameters such as effective population size (*N*_e_) and population density (circular array of 1000 demes each separated by 1 km and with an with Ne of 1000; Kinlan & Gaines, 2003). This dispersal model allows for inferences about the spatial scales over which individuals successfully disperse and establish on a per generational basis and therefore provides information on demographic connectivity, a concept important in fisheries management (Palumbi, 2003). Of further relevance to fisheries management is that IBD slopes, unlike *F*_ST_, are not affected by rare dispersal events but strongly reflect dispersal over the most proximate generations and therefore over ecologically significant time scales (Rousset, 1997).

### 2.7 Connectivity barriers

**FIGURE 2:**
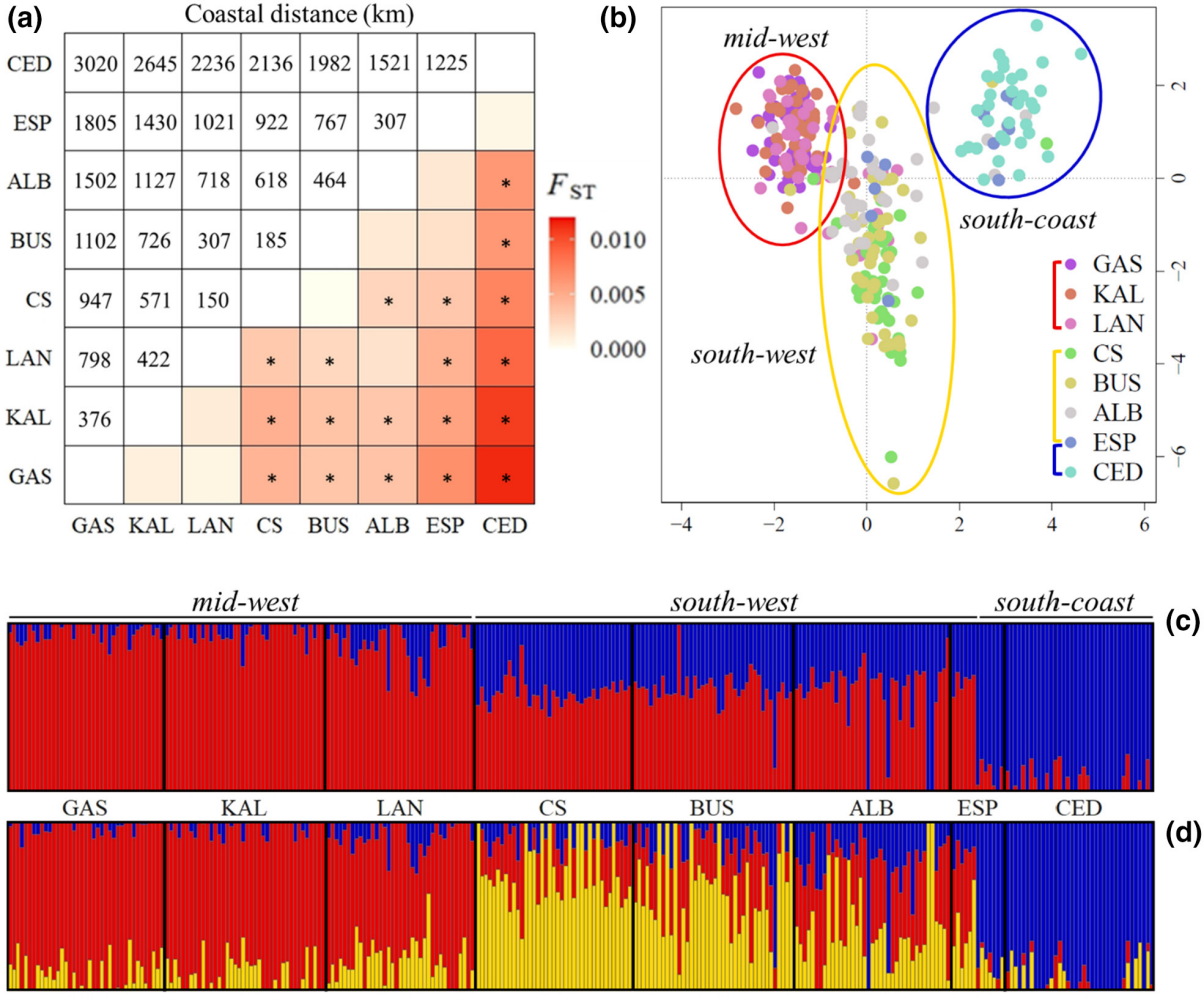
Pairwise estimates of genetic differentiation (a) and broad‐scale genetic clusters (b–d) for snapper between the central west coast of Western Australia (GAS) and the west coast of South Australia (CED). These results are based on the 10,903 neutral SNPs and exclude the two temporal samples. More specifically: (a) a heatmap of pairwise *F*_ST_ values (below diagonal) and the coastal distances between sampled sites (km; above diagonal), with the asterisks indicating *F*_ST_ values that remained significant (α = 0.05) after correction for multiple comparisons via FDR estimation. (b) Principal component analysis (PCA) of genetic differentiation, with principal component 1 (PC1: 0.87% of variance) against principal component 2 (PC2: 0.59% of variance) and each point representing an individual, colour coded by sampling location, and the circles indicating the three identified genetic clusters—the mid‐west (GAS, KAL, LAN), the south‐west (CS, BUS, ALB) and the south‐coast (CED). Note that the ESP sample spans the south‐west and south‐coast groups. Bar plots of the ADMIXTURE clustering analysis results for (c) *K* = 2 and (d) *K *= 3. Labels above the plots represent the three groups identified with the PCA. Individuals are represented by the vertical bars and each individual is coloured according to its probability of membership to each of the three clusters, which are represented by red, yellow and blue

We tested for putative barriers to dispersal using the piecewise regression approach implemented in Robinet et al. (2020) to identify regions where gene flow deviates from an IBD pattern. Briefly, the individual ancestry proportions generated by ADMIXTURE for a *K* of 2 (presented in Figure 2c) were used to estimate a mean SA ancestry (i.e. ancestry characteristic of the site Ceduna) per locality. We then performed a piecewise regression of SA ancestry as a function of distance to Esperance. A barrier to dispersal (i.e. where gene flow is lower than expected under IBD) was considered present when the model provided a significant reduction in the residual sum of squares relative to the simple regression model. For this analysis, we used the R script Introgression_breaks.R (https://github.com/tonyrobinet/introgression).

### 2.8 Local‐scale structure

To investigate local‐scale patterns of gene flow and further explore the impact of coastal distance on genetic structure, we assessed the genetic similarity between individuals at increasing geographic distances with spatial autocorrelation analyses in GENALEX 6.5 (Peakall & Smouse, 2012; Smouse & Peakall, 1999). Spatial autocorrelation analysis can provide higher resolution information on current patterns of gene flow than evolutionary estimators like *F*_ST_ (Peakall et al., 2003). Specifically, the analysis can uncover IBD signals over smaller scales and demarcate ecologically important genetic patches. The extent of nonrandom and positive spatial autocorrelation can be inferred from the first x‐intercept in the correlogram (also referred to as genetic patch size) if a significant correlation coefficient (*r*) occurs in at least one distance class (Smouse & Peakall, 1999; Sokal & Wartenberg, 1983). Analyses were done separately for the two main WA groups identified with the clustering and piecewise regression analyses (i.e. the mid‐west and south‐west groups; see Section 3). Distance classes were chosen such that sample sizes per class were adequate and similar (these were between 1200 and 2300, with only the smallest distance classes involving >2000 comparisons, which occurred because of the large number of individuals landed at the same location). Spatial autocorrelation coefficients were also calculated for all eight samples separately to assess within‐location autocorrelation. The significance of each *r* value was determined with 1000 bootstraps, while 95% CIs around the null hypothesis of randomly distributed genotypes were determined with 1000 permutations. To reduce the possibility of overinterpreting the correlograms, we only considered a value of *r* to be significant if it fell outside of the CIs around the null hypothesis of zero correlation and if its error bars did not cross the *x*‐axis.

## 3 RESULTS

### 3.1 SNP genotyping

A total of 7,342,804 raw variants were identified. After completing all filtering steps and removing candidate adaptive SNPs, our final ddRADseq dataset comprised 10,903 putatively neutral SNPs (details in Table S2). Four of the 345 sequenced samples were removed from the dataset due to having >20% missing data, leaving 341 snapper for subsequent analyses (Table 1). The 341 samples had an average of 1.1% missing data (range: 0.009%–14.1%) and an average coverage depth per locus per sample of 98.8 (range: 3.2–222.6).

### 3.2 Genetic diversity, population differentiation and clustering analyses

Levels of genetic diversity were very similar across sites (Table 1). Expected heterozygosity (*H*_E_) ranged from 0.182 to 0.188 and observed heterozygosity (*H*_O_) ranged from 0.180 to 0.189. Values of the population‐specific inbreeding coefficient (*F*_IS_) were close to zero for all sites (range: 0.005–0.03) and none deviated significantly from expectation.

Genetic differentiation (*F*_ST_) between pairs of sampled sites was nil to low and ranged from 0 to 0.011, with CED being the most differentiated sample (Figure 2a). Despite the low differentiation, 18 of the 28 pairwise site comparisons remained significant after correction for multiple comparisons via FDR estimation. Global *F*_ST_ for the species between the central west‐coast of WA (GAS) and the west‐coast of SA (CED) was 0.0041 (95% CIs: 0.0021, 0.0060).

The PCA indicated the presence of three geographically distinct groups across the study region, referred to herein as the mid‐west (GAS, KAL, LAN), south‐west (CS, BUS, ALB) and south‐coast (CED; Figure 2b). The ESP sample did not cluster predominately with any one group but spanned across the south‐west and south‐coast. Differentiation between the groups varied, with greater distinction observed between the south‐coast and the south‐west and mid‐west groups than between the mid‐west and south‐west groups. Of the two WA groups, the mid‐west was more tightly clustered than the south‐west, suggesting greater homogeneity in the former. The DAPC results were consistent with these PCA outcomes (Figure S1).

The ADMIXTURE analysis suggested that the most probable number of genetic clusters in the dataset was two (i.e. *K* = 2). These clusters loosely corresponded to the samples between GAS and ALB (plus ~ half of the ESP sample) and the South Australia (SA) sample (CED; plus the other half of the ESP sample; Figure 2c). However, inspection of the *q* values for *K* = 2 indicated the presence of two weakly differentiated groups within the larger cluster with clearly distinct ancestry proportions. These groups corresponded with the mid‐west and south‐west clusters identified with the PCA. The ancestry proportions for *K* = 3 distinguished these groups and provided further information on population structure across the study region (Figure 2d). For example, they indicated greater homogeneity in ancestry proportions within the mid‐west than the south‐west group, suggesting that biologically relevant fine‐scale structure may occur within the latter. The results of the analysis excluding CED were consistent with those involving all individuals except that the uniqueness of the ESP sample was lost (i.e. the SA‐like ancestry; Figure S2), highlighting the benefit of including the SA locality in our study.

The AMOVA indicated that 0.43%, 0.07% and 97.3% of the genetic variation in the dataset occurred between the three groups (i.e. the mid‐west, south‐west and south‐coast), among samples within groups and within samples (i.e. the eight samples corresponding to sampling locations), respectively. For this analysis, ESP was allocated to the south‐coast group since the lowest *F*_ST_ involving ESP was with CED (*F*_ST_ = 0.0004, compared to the next lowest estimate *F*_ST_ = 0.0014 with ALB).

**FIGURE 3:**
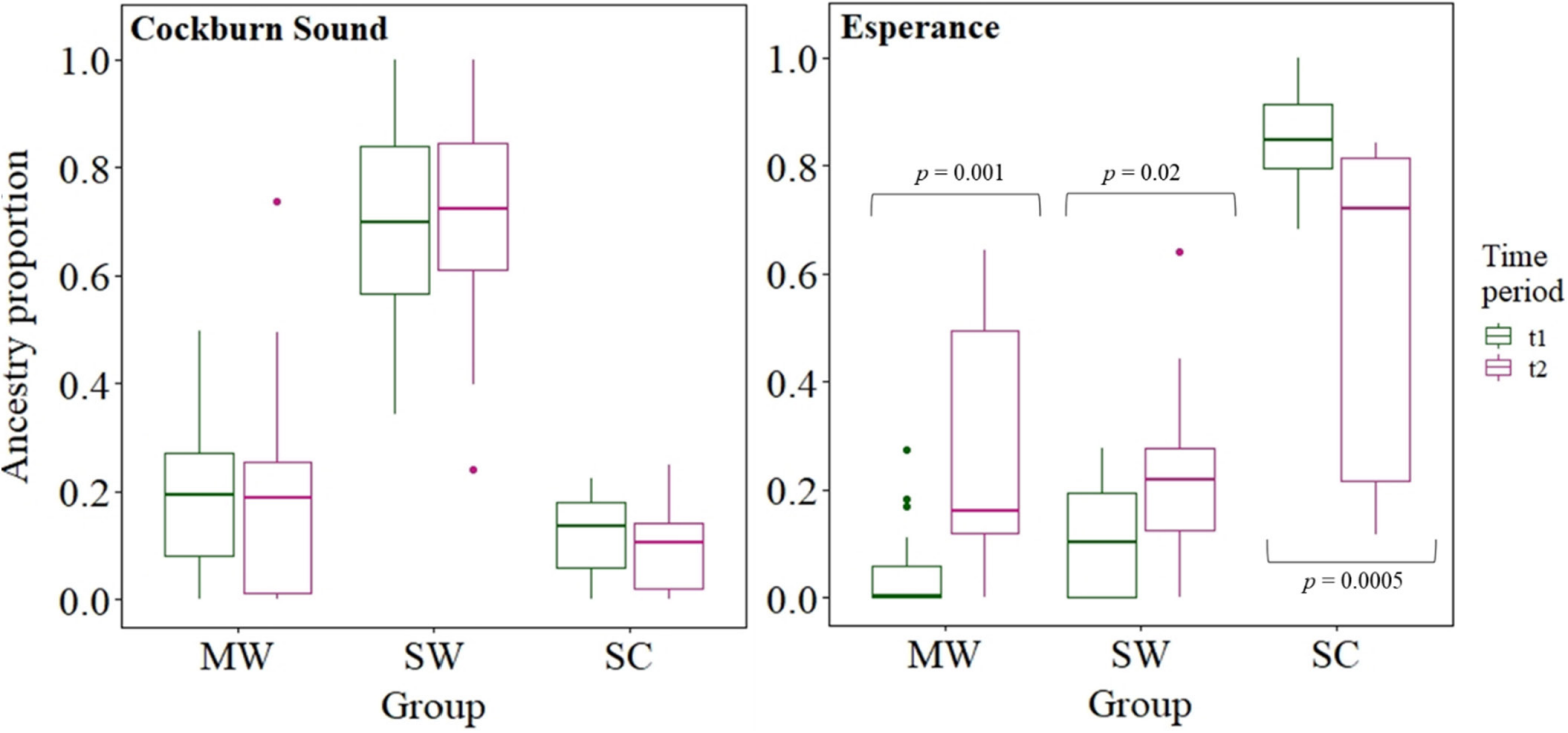
Summary of membership to the three identified groups (i.e. MW, mid‐west; SW, south‐west; SC, south‐coast) for the temporally separated snapper samples from CS and ESP. Ancestry membership proportions did not significantly differ between the two CS samples, but differed for all three comparisons for the ESP samples. Ancestry proportions are from the ADMIXTURE results for *K* = 3. For CS, t1 = 2014 and t2 = 2018, while for ESP, t1 = 2010 and t2 = 2019/2020

Comparing the two temporal CS and ESP samples, membership to the three groups identified with ADMIXTURE indicated temporal stability in genetic structure at the former location only (Figure 3). Compared with the ESP sample, ESP10 had higher membership to the south‐coast group (Wilcoxon test: *p*‐value < 0.0005) and lower membership to the mid‐west and south‐west groups (Wilcoxon test: *p*‐value = 0.001 and 0.02, respectively).

### 3.3 Isolation by coastal distance

**FIGURE 4:**
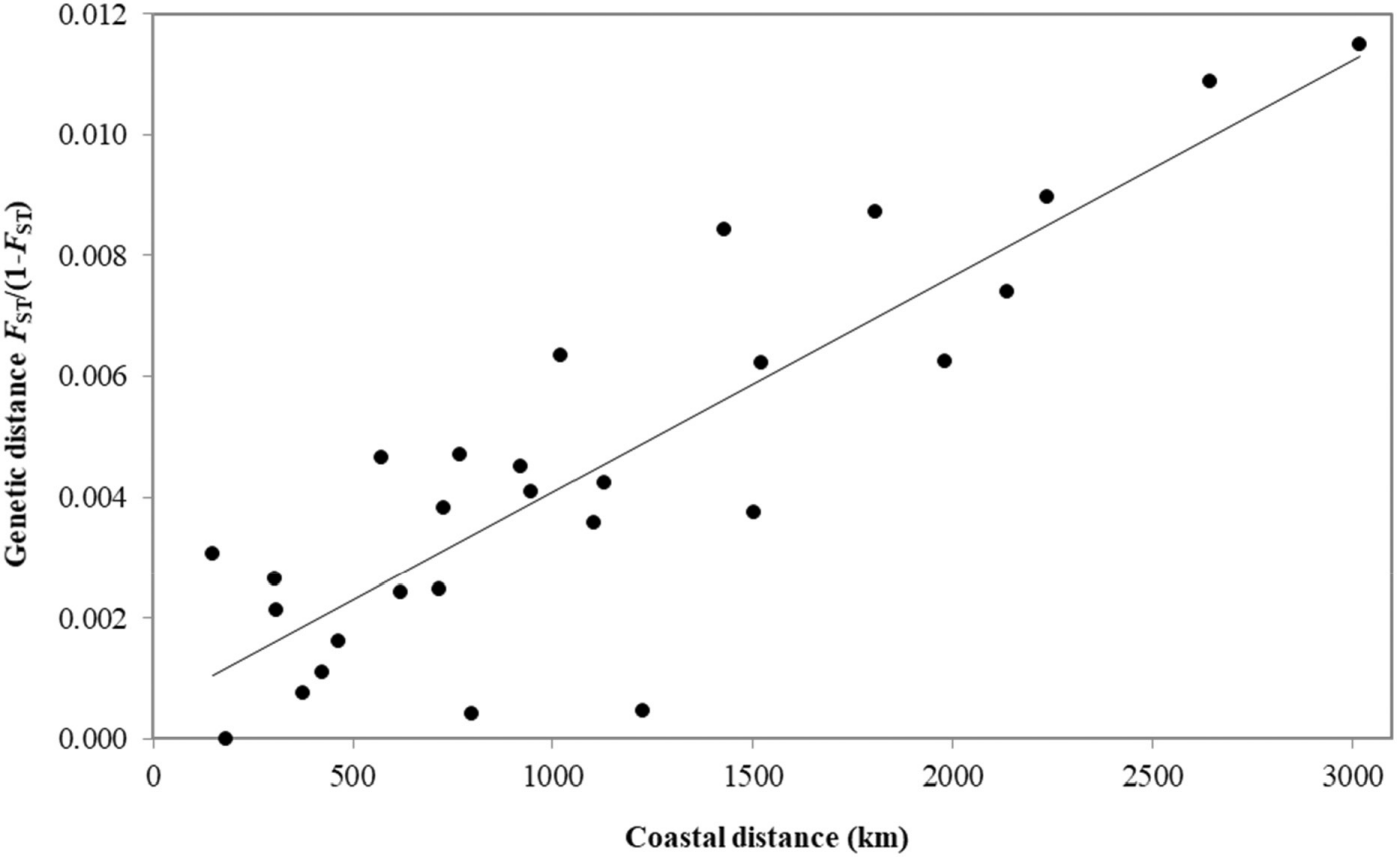
Relationship between coastal distance and genetic distance (linearized *F*_ST_) for snapper between the central west coast of Western Australia (GAS) and the west coast of South Australia (CED; Mantel test: *r* = 0.86, *p*‐value <0.001)

We detected highly significant IBD across the sampling range (*r* = 0.86, *p*‐value <0.001; Figure 4). This analysis indicated that spatial distance explains 74% of the variation in linearized *F*_ST,_ accounting for a substantial amount of the population genetic differentiation inferred across the sampling range. The mean effective dispersal distance per generation was estimated as 400 km from an IBD slope of 4E‐06 (i.e. *F*_ST_ = 0.004 per 1000 km). This suggests that demographic connectivity between locations separated by distances greater than 400 km may be limited. Our estimate of 400 km is within the range of those made for other marine fish (Kinlan & Gaines, 2003).

### 3.4 Barriers to connectivity

**FIGURE 5:**
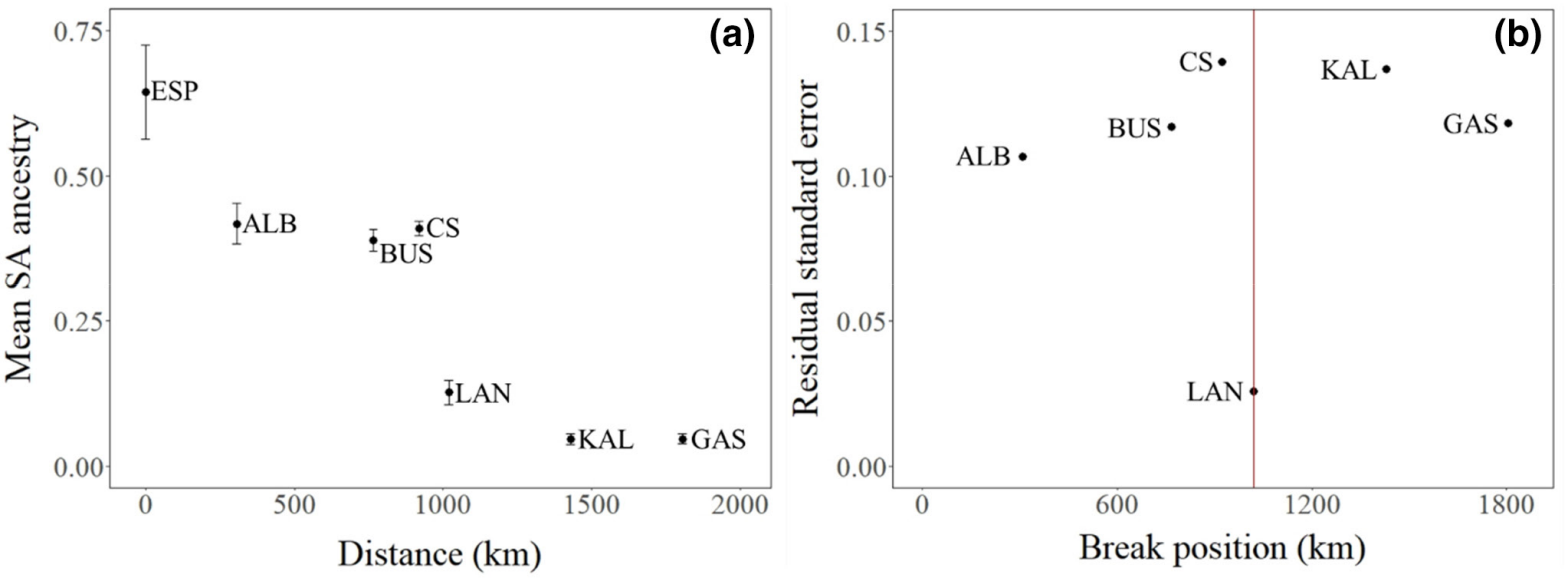
Analysis of barriers to connectivity in snapper along the Western Australia (WA) coast. (a) Mean proportion of South Australian (SA) ancestry (i.e. ancestry characteristic of the blue group CED in Figure 2c) across the WA samples (±standard error), determined from the ADMIXTURE results for *K* = 2, as a function of distance from ESP, and (b) results of the piecewise regression analysis showing residual standard error as a function of distance from ESP

The piecewise regression analysis identified a significant breakpoint (i.e. where gene flow is lower than would be expected under IBD) in the ancestry gradient between CS and LAN, despite these sites being separated by only ~150 km (Figure 5). Although the simple linear regression model revealed a strong negative relationship between distance and mean SA ancestry (adj. *r* ^2^ = 0.67, *p*‐value = 0.03), the piecewise regression model was a significantly better fit to the data (piecewise model: adj. *r* ^2^ = 0.98, *p*‐value = 0.01; ANOVA for model comparison: *p*‐value < 0.001). These results suggest that the genetic structure observed along the WA coast is not merely due to IBD but is also a reflection of a connectivity barrier, an interpretation consistent with the results of the clustering analyses.

### 3.5 Local‐scale structure

**FIGURE 6:**
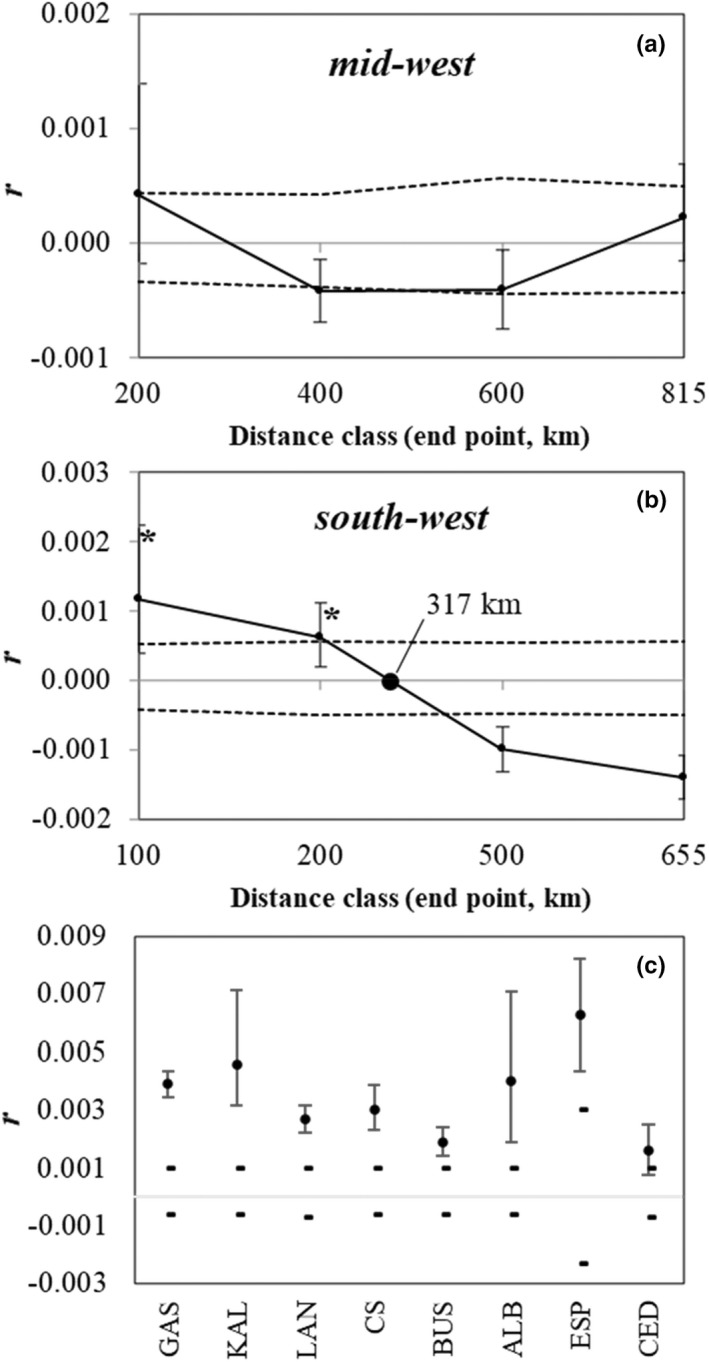
Spatial autocorrelation analyses of snapper samples from the (a) mid‐west, (b) south‐west and (c) each of the eight samples separately. These analyses exclude the two temporal samples and are based on 10,903 neutral SNPs. Values of *r* represent genetic autocorrelation coefficients, the dashed lines represent 95% CIs around the null hypothesis of randomly distributed genotypes, determined with 1000 permutations, and the error bars represent 95% CIs around *r* values, as determined by 1000 bootstraps. In the correlograms, the first *x*‐intercept following significantly positive values of *r* (denoted by asterisks) indicates the extent of positive spatial autocorrelation or the genetic patch size

The spatial autocorrelation analyses indicated that across the sampling range, positive genetic autocorrelation occurs between individuals sampled at the same site (Figure 6c), a result suggestive of recruitment to the local subpopulation. Within site spatial autocorrelation was highest at GAS and KAL in the mid‐west and at ALB in the south‐west (Figure 6c). Variation around sample‐specific *r* values was highest for KAL, ALB and ESP. These samples were the result of the greatest number of fishing days that were separated by the greatest stretches of time (Table S1). This could perhaps indicate that cohesion among different groups of genetically alike individuals may occur in snapper across this region, but such a possibility would need to be verified with larger samples than those available here.

Our spatial autocorrelation correlograms indicated that local‐scale genetic structure occurs within the south‐west but not the mid‐west (Figure 6a,b), a result concordant with observations made from the PCA and ADMIXTURE plots. In the mid‐west, genetic autocorrelation occurred only among individuals landed at the same site, suggesting that it is largely a homogenous group (Figure 6a,c). In contrast, in the south‐west, significant positive spatial autocorrelation also occurred in the 100–200 km distance class (Figure 6b). The shape of the correlogram was of the ‘long‐distance cline’ type (Diniz‐Filho & De Campos Telles, 2002; Östman et al., 2017), suggesting that IBD occurs across the south‐west. The south‐west had an x‐intercept and therefore a genetic patch size of ~300 km, indicating the scale over which high levels of gene flow occur. This estimate also suggests that in the south‐west, individuals separated by >300 km are less genetically similar than expected by chance.

## 4 DISCUSSION

This is the first study to investigate population structure and connectivity in the fishery important Australian snapper using a population genomics approach. We detected spatially variable population genetic structure and levels of connectivity in snapper across ~3000 km of coastline. Although our results indicated that snapper across Western Australia (WA) are characterized by weak genetic structure, we found evidence for stock discontinuities, dispersal limits and local‐scale structure. Our results improve upon prior understanding of population structure and connectivity in snapper across its WA range based on tagging (Crisafulli et al., 2019; Wakefield et al., 2011), otolith microchemistry (Edmonds et al., 1999; Fairclough et al., 2013) and microsatellite analyses (Gardner & Chaplin, 2011). They also partly contrast with the spatial scales of assessment and management currently used for snapper in WA (see Fairclough et al., 2021), indicating that a review may be beneficial. Our study demonstrates the value of population genomic surveys to inform the management of weakly structured and wide‐ranging marine fishery resources.

### 4.1 Broad‐scale structure

We uncovered two broad‐scale patterns of population genetic structure in snapper across WA with our neutral SNP dataset. Firstly, strong isolation by distance (IBD) was detected between the central west‐coast of WA and the west‐coast of South Australia (SA), indicating that dispersal is limited by coastal distance and probably primarily occurs in a stepping‐stone fashion. Previous work with microsatellite markers and otolith microchemistry also uncovered IBD in snapper across WA (Edmonds et al., 1999; Fairclough et al., 2013; Gardner & Chaplin, 2011). IBD has also been detected in a number of other broadcast spawning marine species within our study range, including Roe's abalone (Hancock, 2000), WA dhufish (Berry et al., 2012) and saucer scallop (Kangas et al., 2019).

Broad‐scale IBD signals do not convey information on whether genetic discontinuities exist or where they are located. The clustering and connectivity barriers analyses allowed us to detect two genetic discontinuities, lying on the lower west coast and in the southeast of the study range. Interestingly, both boundaries are not marked by obvious physical barriers. This is a common finding in population genomic studies on marine species (Alberto Mares‐Mayagoitia et al., 2021; Deli et al., 2020; Portnoy et al., 2022; Silliman, 2019; Xuereb et al., 2018) and points to the complexity of evolutionary processes that maintain population structure. Although Gardner and Chaplin (2011) also detected the discontinuity in the southeast using microsatellite markers, our study is the first to report a stock boundary for snapper on the lower west coast. Otolith microchemistry work with snapper along the west coast of WA uncovered differentiation between snapper north and south of the Abrolhos Islands, located on the mid‐west coast (Figure 1; Edmonds et al., 1999). However, that study lacked samples from locations between the Abrolhos Islands and Cockburn Sound so could not have detected a discontinuity just south of Lancelin. Additionally, although another otolith microchemistry study detected differentiation in snapper along the west coast of WA, a clear stock boundary was not detected between Cockburn Sound and Lancelin, potentially due to the often weak microchemistry differences in marine environments (Fairclough et al., 2013). Our study has therefore generated the clearest and most detailed account yet of the stock structure and connectivity of snapper in WA.

Generally, the stock discontinuities lie between locations within our study's range where the largest aggregations of spawning snapper have been observed – the Shark Bay region, Cockburn Sound (including the adjacent embayments Owen Anchorage and Warnbro Sound) and the SA gulfs (Spencer Gulf and Gulf St Vincent) – and therefore may indicate the importance of these spawning areas in maintaining spatially proximate snapper stocks. The discontinuities identified here delineate three broad‐scale snapper stocks (i.e. the mid‐west, south‐west and south‐coast stocks; see Figure 2). The size and spatial boundaries of the three snapper stocks are probably largely shaped by spawning timing and location, as well as oceanography and coastal topography. The mid‐west stock is likely the larger of the two purely WA stocks because spawning grounds in the Shark Bay region are exposed to the Shark Bay Outflow (see Figure 1; Hetzel et al., 2018) and a strong Leeuwin Current (LC; Cresswell & Golding, 1980, Godfrey & Ridgway, 1985), facilitating the southward transport of pelagic life stages over potentially several hundreds of kilometres (Feng et al., 2010). In contrast, in the south‐west, the magnitude of dispersal of PLS snapper from Cockburn Sound is probably limited because it is largely protected from shelf currents like the LC and Capes Current (see Figure 1; Pearce & Pattiaratchi, 1999). Instead, wind‐driven gyres within the embayment (Steedman & Craig, 1983) facilitate the retention of eggs and larvae (Wakefield, 2010), limiting long‐distance transport of PLS.

The boundary between the mid‐west and south‐west stocks lies between ecological bioregions (inshore: Southwest Shelf Transition and Southwest Shelf Province, offshore: Central Western Transition and Southwest Transition) delineated from patterns of species distributions and environmental features (Commonwealth of Australia, 2006). The boundary between these bioregions coincides with patterns in the distribution of invertebrates (Kott, 1952; O'Hara & Poore, 2000), fishes (Ayvazian & Hyndes, 1995; Hutchins, 1994; Last et al., 2011; Williams et al., 2001) and seaweeds (Wernberg et al., 2013). These studies, along with ours, indicate that there may be oceanographic features in the region capable of disrupting alongshore transport of PLS. Indeed, the marine environment around Lancelin is dynamic and comprises features that could achieve this. South of the Abrolhos Islands (Figure 1) the LC strengthens, leading to its instability and the production of eddies just north of Lancelin where the shelf narrows, causing the offshore movement of water (Feng et al., 2007; Pattiaratchi, 2006; Pearce & Griffiths, 1991). Circulation patterns off Lancelin, along with the retention capability of the gyres in Cockburn Sound, may contribute to the stock boundary observed for snapper between the mid‐west and south‐west regions.

In contrast to the break between the mid‐west and south‐west stocks, a transition region occurred between the south‐west and south‐coast stocks. The heterogeneous Esperance sample occurred within this transition region, with approximately half of the sample having south‐west ancestry and the other half having south‐coast ancestry. The location of this transition region may in part reflect the magnitude of dispersal events from the SA gulfs (relative to those from further west). It is likely that this westward dispersal occurs post‐recruitment, since thermal fronts form at the entrances of the SA gulfs during the spawning season, largely preventing gulf‐shelf exchanges (Bruce & Short, 1990; Petrusevics, 1993; Vaz et al., 1990). For example, in *Nerita atramentosa* (an intertidal snail with a pelagic larval duration of around four months), the percentage of larvae released during summer in the two SA gulfs that did not reach the boundary current ranged from 70% to 100% (Teske et al., 2015). Hypothetically, long‐distance dispersal from the SA gulfs may occur during the subadult stage, a conclusion of otolith microchemistry work (Fowler et al., 2005). We however acknowledge that the large sampling gap between Esperance and Ceduna (~1200 km) means that we cannot characterize the exact nature of the discontinuity between the south‐west and south‐coast stocks.

### 4.2 Local‐scale structure

The strength of an IBD relationship may be greater in some areas and weaker in others. The spatial autocorrelation analyses allowed us to investigate whether local‐scale patterns of genetic structure in WA mirror the broad‐scale signal of IBD. Our results indicated that the mid‐west deviates from the broad‐scale IBD pattern and constitutes a well‐mixed stock of snapper, which is likely facilitated by the LC (flowing strongly during the winter, the mid‐west spawning season). In contrast, in the south‐west, we detected a pattern of spatial autocorrelation consistent with IBD, potentially facilitated by a weak LC during snapper's spring/summer spawning period in the region and the location of important spawning sites (i.e. protected embayments). Positive spatial autocorrelation occurred at distances up to ~300 km, suggesting that demographic connectivity between our sites on the western and southern coastlines of the region is probably limited and may occur in a stepping‐stone fashion. King George Sound in Albany is an important spawning area for snapper along the south coast of WA (Wakefield et al., 2015). It is possible that this embayment, along with a number of smaller adjacent embayments, is most important for supplying parts of the southern coastline of the south‐west (Neira & Potter, 1992; Potter et al., 1990). Overall, our spatial autocorrelation analyses suggest that the influence of oceanographic processes on the transport of PLS may be particularly important in shaping local‐scale patterns of genetic structure in snapper in WA.

### 4.3 Temporal variability in genetic structure

Analysis of the two temporally replicated samples uncovered differences in ancestry membership to the three identified stocks at Esperance, but not at Cockburn Sound. Temporal variation in population genetic structure has been reported for numerous marine species (Hogan et al., 2010; Jackson et al., 2018; Papetti et al., 2009; Quintero‐Galvis et al., 2020; Watts et al., 2010). When comparing the repeated Esperance samples, there was a significant reduction in ancestry characteristic of our SA sample over time and an increased contribution from stocks further west (i.e. mid‐west and south‐west). This shift suggests that the genetic structure of snapper off Esperance may largely depend on dynamics of snapper populations in SA and along the south coast of WA, including year‐to‐year recruitment strength. Indeed, interannual recruitment variability can be substantial in snapper and is considered to greatly influence population dynamics (Fowler & McGlennon, 2011; Francis, 1993; Hamer & Jenkins, 2004; Wakefield et al., 2015). Recent stock depletions and prolonged recruitment failure in the SA gulfs may account for the change in population genetic structure at Esperance (Fowler et al., 2020). The shift in genetic composition of Esperance snapper also implies that the location of the genetic discontinuity in the south‐east varies temporally. Overall, our temporal analyses indicate that incorporating samples collected at multiple timepoints in population genetic studies can reveal important biological information that may have otherwise gone undetected.

### 4.4 Suitability of current stock boundaries based on population genomics

Employing a large genome‐wide dataset gave us sufficient power to uncover patterns of population genetic structure and connectivity at multiple spatial scales. We can therefore confidently use our findings to inform the spatial assessment and management of snapper. The patterns of genetic structure in snapper uncovered in this study partly contrast with current spatial scales of stock assessment and management. Across WA (outside of the inner gulfs of Shark Bay), three snapper stocks are currently recognized for assessment and management: the Shark Bay Oceanic (comprising Gascoyne), West Coast (comprising Kalbarri, Lancelin, Cockburn Sound and Busselton) and South Coast stocks (comprising Albany and Esperance; see Figure 1). For the West Coast, a whole of bioregion assessment is conducted along with two smaller area‐based assessments, with the first including locations south of Lancelin and the second locations north of Lancelin to Kalbarri. The most recent assessment identified differences in stock status between these areas, related to variation in levels of exploitation (Fairclough et al., 2021). There are also known differences in biological characteristics (e.g. length and age) between the southern west coast and the central west coast (e.g. see Figure 1 inset). Consistent with this, we detected a stock boundary between Lancelin and Cockburn Sound, as well as a lack of genetic structure over an area extending ~800 km between the Gascoyne and Lancelin. Our results therefore suggest that managing the mid‐west stock separately may be beneficial. However, such an approach would need to consider the fact that the fisheries that operate in the region are multispecies and multisector and that other species may have differing population structuring.

In contrast to the mid‐west, in the south‐west of WA between Cockburn Sound and Albany, local‐scale genetic structure was uncovered whereby genetic differentiation increased with coastal distance (i.e. an IBD pattern). Simulation work by Spies et al. (2015) showed that splitting an area characterized by IBD for management purposes leads to more favourable outcomes than if considered singularly (e.g. spawning biomass). Further, they demonstrated that the most optimal outcomes could be achieved by splitting a management region according to dispersal distance and fishing effort. Others suggest that under a pattern of IBD, spatial scales of management might be based on the x‐intercept of spatial autocorrelation correlograms, a value that indicates the distance at which individuals become genetically independent (Diniz‐Filho & De Campos Telles, 2002; Östman et al., 2017). Considering our estimate of mean dispersal distance per generation determined from the IBD slope (~400 km), the x‐intercept of our spatial autocorrelation correlogram (~300 km) and that fishing pressure is greater along the west coast near the Perth metropolitan region than the south coast (Gaughan & Santoro, 2021), the current management boundary on the south‐west corner of the state (at 115.5°E, see Figure 1) appears suitable.

Along the south coast of WA, the region between the south‐west corner and the SA border (i.e. the south‐coast stock) is assessed and managed as a single unit (Newman et al., 2022). Here we detected significant gene flow across this region. However, the genetic composition of snapper in the eastern part of the region (i.e. at Esperance) varies temporally and therefore the location of the boundary between SA and WA snapper changes over time. Considering this finding, and that catch data indicate that the biomass of snapper in the south east of WA is likely low relative to adjacent areas (Norriss et al., 2016), it may be appropriate to consider the eastern part of the southern coast of WA (i.e. east of Albany) separately for stock assessment and management purposes. The southern coast of WA presents a more complex situation for spatial assessment and management than the west coast and highlights the difficulties in translating complex biological processes into distinct units suitable for fisheries management. Population genomic work involving finer scale sampling along the southern coast of WA and the west coast of SA (i.e. between Albany and Ceduna) would be valuable for discerning the most suitable locations for drawing management boundaries. Additionally, our temporal analysis suggests that regular genetic monitoring would be beneficial to better understand the temporal and spatial dynamics of the stock boundaries uncovered here.

## 5 CONCLUSIONS

The fluidity of the marine environment, along with the highly mobile and abundant nature of the species which occupy it, makes the characterization of evolutionary and demographic patterns in marine organisms difficult. Our study highlights the utility of large datasets of genetic markers in improving the understanding of spatial population structure and connectivity in marine species with high dispersal potential. Our findings likely relate to variation in physical factors, such as local ocean circulation and coastal geomorphology, as well as in biological factors, including timing of reproduction, spawning site preferences, recruitment variability and migratory behaviour. Overall, our study demonstrates the value of population genomics in helping to improve the spatial management of fishery resources and therefore their long‐term sustainability. Additionally, our work adds to the extensive body of literature showing that marine species are often characterized by complex population structure, rather than panmixia, and that population structure is not always static across time.

## CONFLICT OF INTEREST

The authors declare no conflict of interest.

## Supporting information


Appendix S1

## Data Availability

The SNP dataset is available on figshare: https://doi.org/10.6084/m9.figshare.19738651.v1.
