# Supplementary material for: Fisheries genomics of snapper (Chrysophrys auratus) along the west Australian coast
Source: Evol Appl. 2022 Jul 9;15(7):1099–114. doi: 10.1111/eva.13439 (PMC9309437; doi:10.1111/eva.13439)
Supplement: Supplementary file 1 — Appendix S1 [file EVA-15-1099-s001.docx]

|  | Jurisdiction | Management area | Avg. latitude | Avg. longitude | Spawning season (peak) | Catch dates | Sector | Sex ratio (M:F) | Gonad stage |
| --- | --- | --- | --- | --- | --- | --- | --- | --- | --- |
| Gascoyne (GAS) | Western Australia | Shark Bay Oceanic | -24.6667 | 113.0833 | May-Sep (Jun/Jul) | Jul 18 | Commercial | 0.6 | 4.0 (0.8) |
| Kalbarri (KAL) | Western Australia | West Coast | -27.8557 | 113.425 | Jul-Oct (Jul/Aug) | Jul-Sep 18 | Commercial/  Research | 1.2 | 3.6 (0.9) |
| Lancelin (LAN) | Western Australia | West Coast | -30.964 | 115.036 | Oct-Dec (Nov/Dec) | Oct 18 | Research | 1.1 | 3.3 (1.0) |
| Cockburn Sound (CS) | Western Australia | West Coast | -32.1855 | 115.7156 | Oct-Dec (Nov/Dec) | Oct 18 | Research | 1.2 | 4.4 (0.5) |
| Cockburn Sound 14 (CS14) | Western Australia | West Coast | -32.194 | 115.7442 | Oct-Dec (Nov/Dec) | Oct 14 | Research | 0.8 | NA |
| Busselton (BUS) | Western Australia | West Coast | -33.6343 | 115.2958 | Oct-Dec (Nov/Dec) | Jul-Aug 18 | Recreational | 1.0 | 2.5 (0.6) |
| Albany (ALB) | Western Australia | South Coast | -35.1815 | 118.3654 | Oct-Dec (Oct/Nov) | Aug-Nov 18 | Commercial | 0.9 | 3.5 (1.1) |
| Esperance (ESP) | Western Australia | South Coast | -34.2402 | 121.3276 | Oct-Dec (Oct/Nov) | Jun-Feb 19/20 | Commercial/  Recreational | NA | NA |
| Esperance 10 (ESP10) | Western Australia | South Coast | NA | NA | Oct-Dec (Oct/Nov) | Mar 10 | Recreational | NA | NA |
| Ceduna (CED) | South Australia | Spencer Gulf/ West Coast | -32.3 | 133.8 | Nov-Feb (Dec) | Sep 18, Aug 19 | Commercial | 1.1 | 1.6 (0.5) |

**TABLE S1** Biological and catch data for each of the 10 snapper samples (i.e., the nine Western Australian samples, including the two temporal samples, and the comparative sample from South Australia) as well as a summary of timing of spawning for each of the sites sampled. Values in the gonad stage column are means and standard deviations (in parentheses). Gonad stages are: 1, immature; 2, resting; 3, developing; 4, developed; 5, spawning; 6, spent.

|  | Jurisdiction | Management area | Avg. latitude | Avg. longitude | Spawning season (peak) | Catch dates | Sector | Sex ratio (M:F) | Gonad stage |
| --- | --- | --- | --- | --- | --- | --- | --- | --- | --- |

**TABLE S2** Numbers of SNPs retained after each bioinformatics filtering step.

| Step | SNP count |
| --- | --- |
| Raw SNP catalogue | 7,342,791 |
| 80% of individuals, biallelic, >0.03 minor allele frequency | 50,129 |
| Remove indels | 45,927 |
| Read quality (ratio quality/coverage depth >0.2) | 45,349 |
| Mapping quality (>30) | 41,455 |
| High coverage SNPs (≤mean depth + (2*standard deviation)) | 40,406 |
| Hardy-Weinberg equilibrium in >67% of locations | 35,689 |
| Call error rate (0.95) | 32,208 |
| Linkage disequilibrium (500) | 11,253 |
| Putatively neutral SNPs | 10,903 |


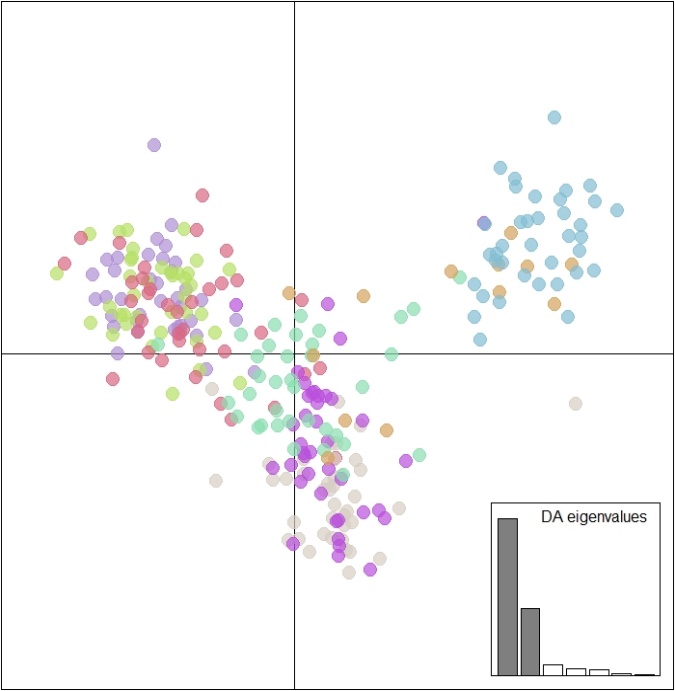

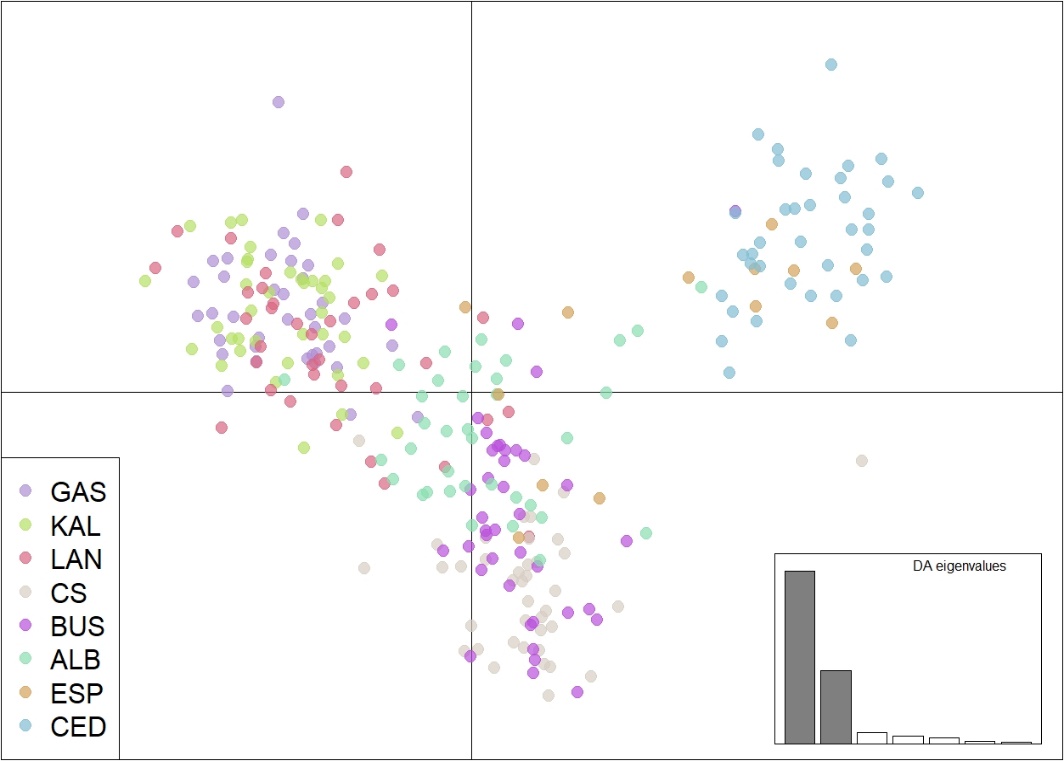


(a)


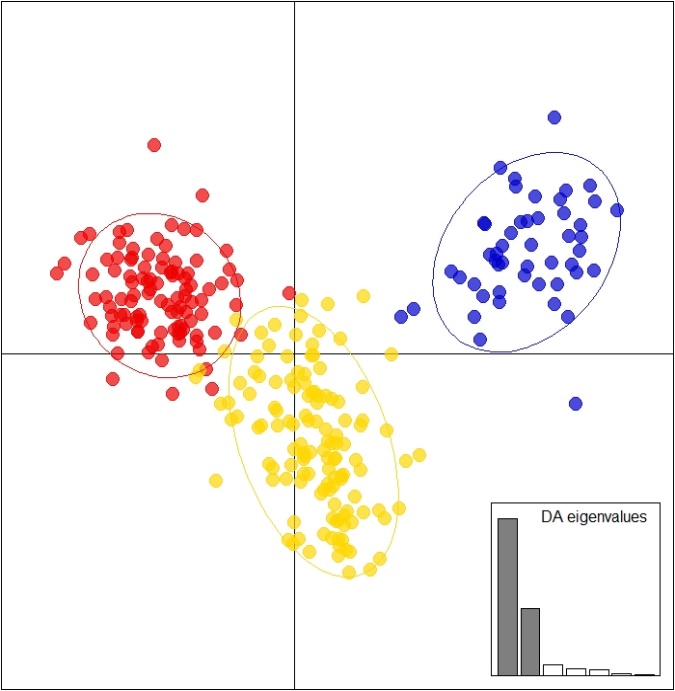

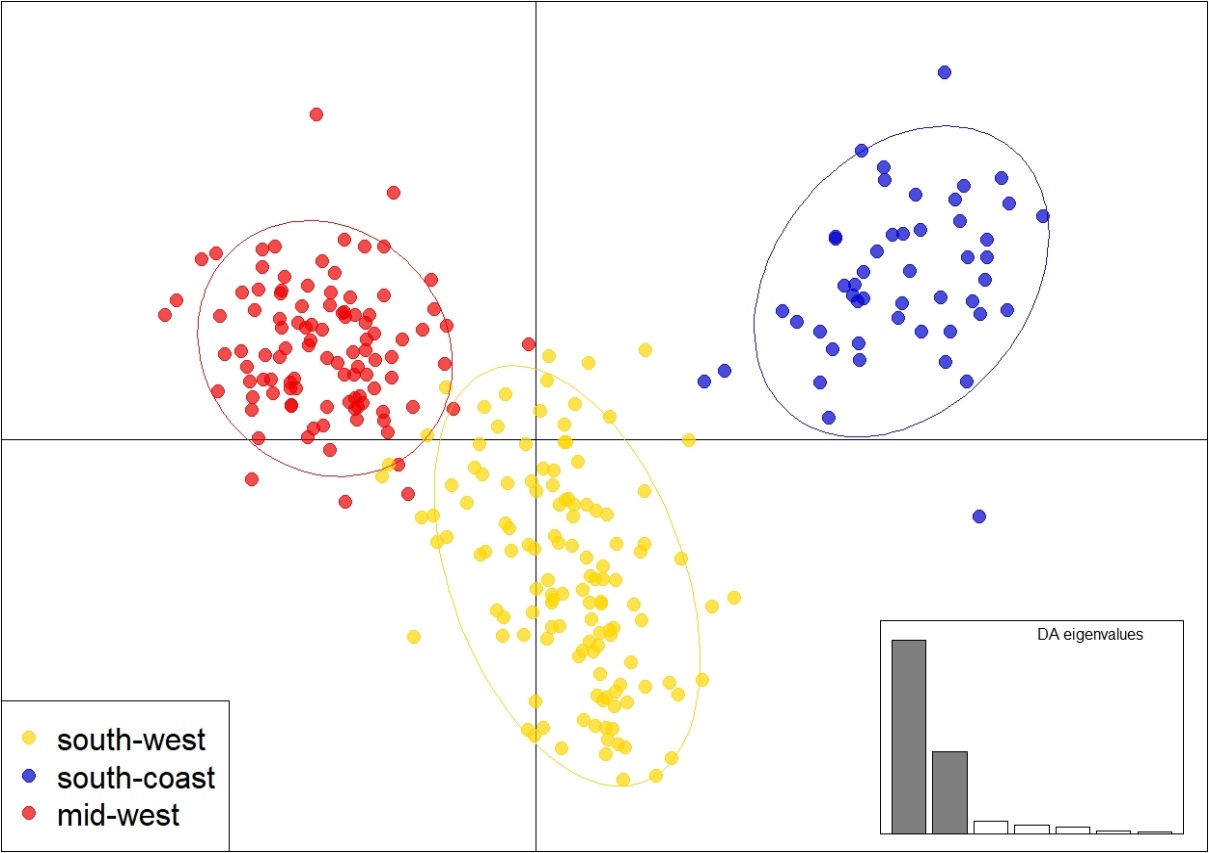

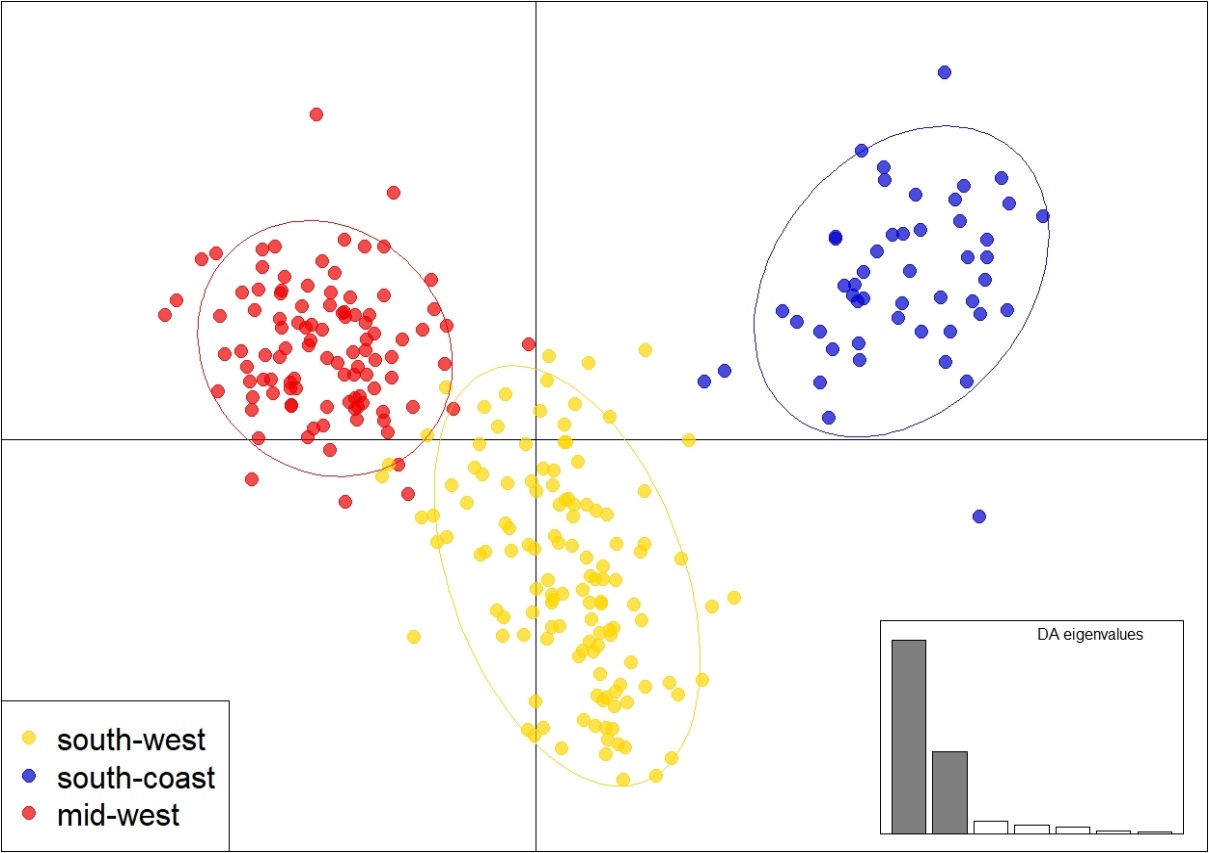


(b)


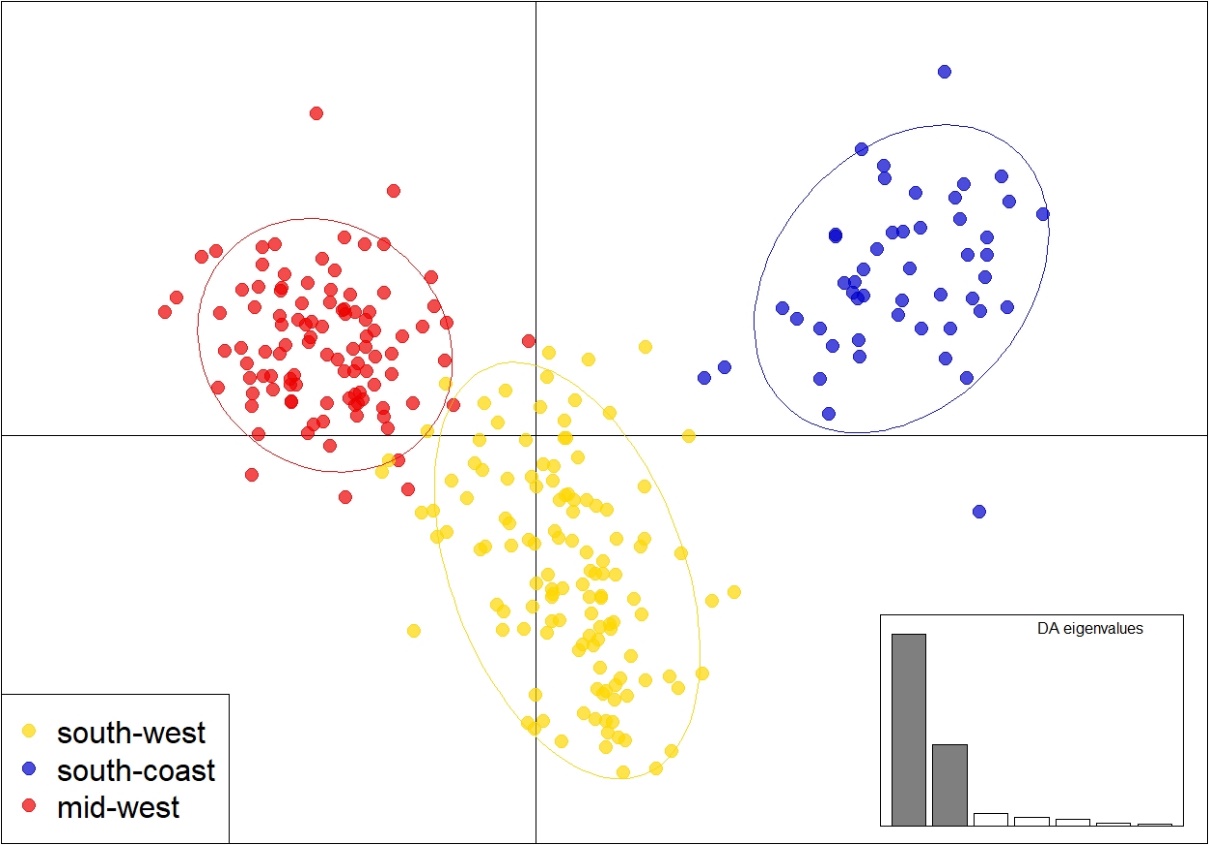


**FIGURE S1** Scatterplots of the results of the discriminant analysis of principal components (DAPC) based on 10,903 SNPs, with points (i.e., individuals) coloured by (a) sampling location or (b) assigned genetic cluster (K = 3). The first two discriminant functions are shown, which explain 62.7% and 26.6% of the genetic variation in the dataset respectively.


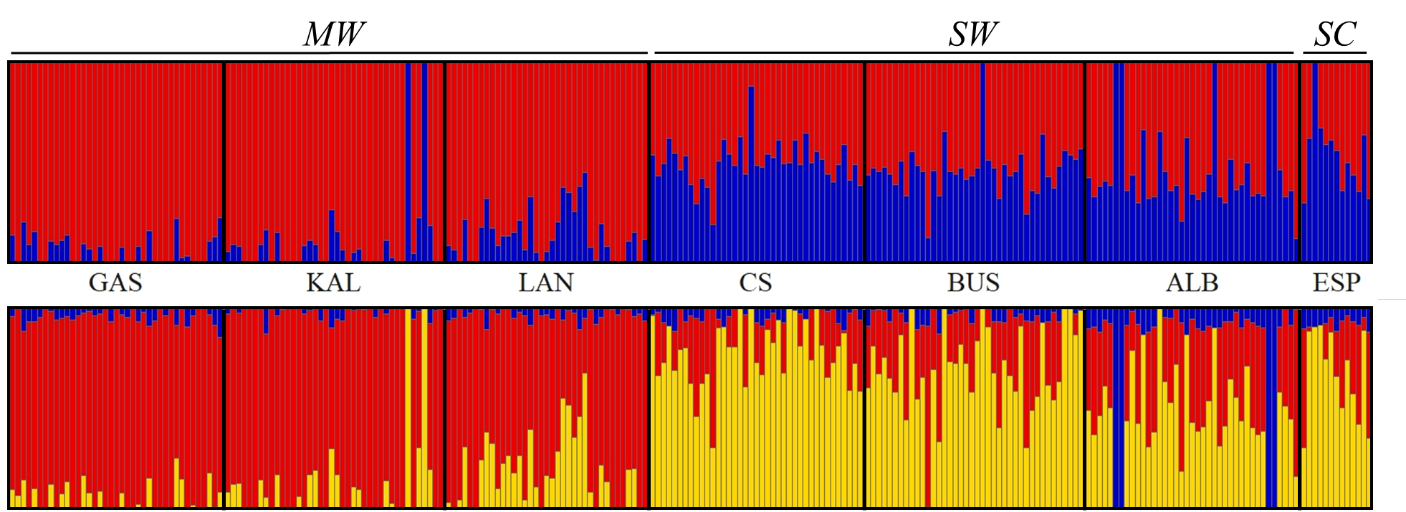


**FIGURE S2** Results of the ADMIXTURE analysis based on the 10,903 SNPs excluding the sample from South Australia (i.e., CED). Plots for K = 2 (above) and K = 3 (below) are shown for comparative purposes (i.e., against Figure 2c and d). Labels above the plots represent the groups identified using the whole dataset (i.e., MW, mid-west; SW, south-west; SC, south-coast).
